# Supplementary material for: No evidence for prolactin’s involvement in the post-ejaculatory refractory period
Source: Commun Biol. 2021 Jan 4;4:10. doi: 10.1038/s42003-020-01570-4 (PMC7782750; doi:10.1038/s42003-020-01570-4)
Supplement: Supplementary file 2 — Description of Additional Supplementary Files [file 42003_2020_1570_MOESM2_ESM.pdf]

Description of Additional Supplementary Files

File name: Supplementary Data 1

Description: Table with the percentage of males that reached ejaculation in all experiments presented in the paper plus one control experiment with unmanipulated animals.

File name: Supplementary Data

Description: Additional analysis of the sexual behavior of males pre-treated with vehicle or Domperidone.

File name: Supplementary Data 3

Description: Analysis of the locomotor activity of bromocriptine treated males.

File name: Supplementary Data 4

Description: Additional analysis of the sexual behavior of males pre-treated with vehicle or Bromocriptine.
