# Supplementary material for: No evidence for prolactin’s involvement in the post-ejaculatory refractory period
Source: Commun Biol. 2021 Jan 4;4:10. doi: 10.1038/s42003-020-01570-4 (PMC7782750; doi:10.1038/s42003-020-01570-4)
Supplement: Supplementary file 3 — Supplementary Data [file 42003_2020_1570_MOESM3_ESM.pdf]

Supplementary Data

No evidence for prolactin's involvement in the post-ejaculatory refractory period

Valente et al.

Supplementary Data 1 Percentage of BL6 males that reached ejaculation.

Comparison of the percentage of BL6 males that ejaculated in the different type of experiments: a) PRL monitoring: tail tip blood collection during the trial; b) PRL manipulation: substance injection prior beginning of the trial; c) un-manipulated males.

**Experiment type**		**# males that ejaculated**	**Total number males**	**% males that ejaculated**
a) PRL monitoring	Fig. 1a-b	8	23	34.8
b) PRL manipulation – Veh	Fig. 2c	13	17	76.5
b) PRL manipulation – Domp	Fig. 2c	11	17	64.7
b) PRL manipulation – Veh	Fig. 3c	15	18	83.3
b) PRL manipulation – Bromo	Fig. 3c	13	18	72.2
c) Un-manipulated	---	6	9	66.7

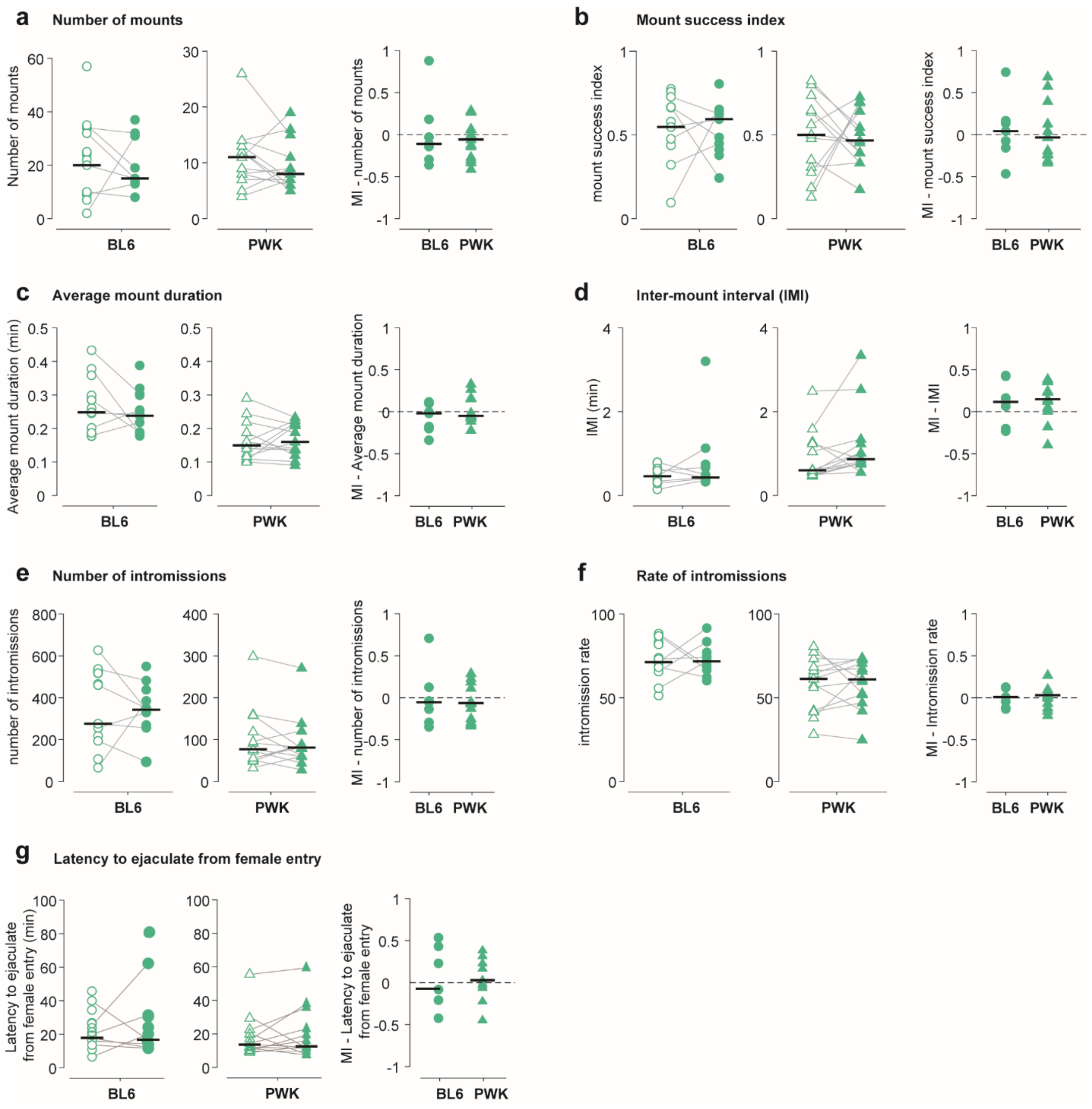

Supplementary Data 2 Additional analysis of the sexual behavior assay using adult sexually trained BL6 and PWK males pre-treated with vehicle or Domperidone (total number of subjects used: BL6 n = 17; PWK, n =13; with ejaculation in both sessions BL6 n = 7; PWK, n =13). Quantification of **a** number of mounts, **b** mount success index [mounts/(mount attempt + mounts)], **c** mount mean duration (total mount duration/#mounts), **d** Inter-mount interval (IMI), **e** number of intromissions, **f** rate of intromissions (#intromissions/total mount duration) and **g** latency to ejaculate from female entry. Each animal underwent two behavioral assays: one with vehicle and one with domperidone. Administration was counterbalanced between animals. Each line represents data of an individual; Unconnected dots represent data of an individual that did not ejaculate in the matching trial (data not used for statistics). MI [modulation index (domp-

veh)/(dom+veh)]. Data presented as median ± M.A.D. (median absolute deviation with standard scale factor) following Wilcoxon rank sum test.

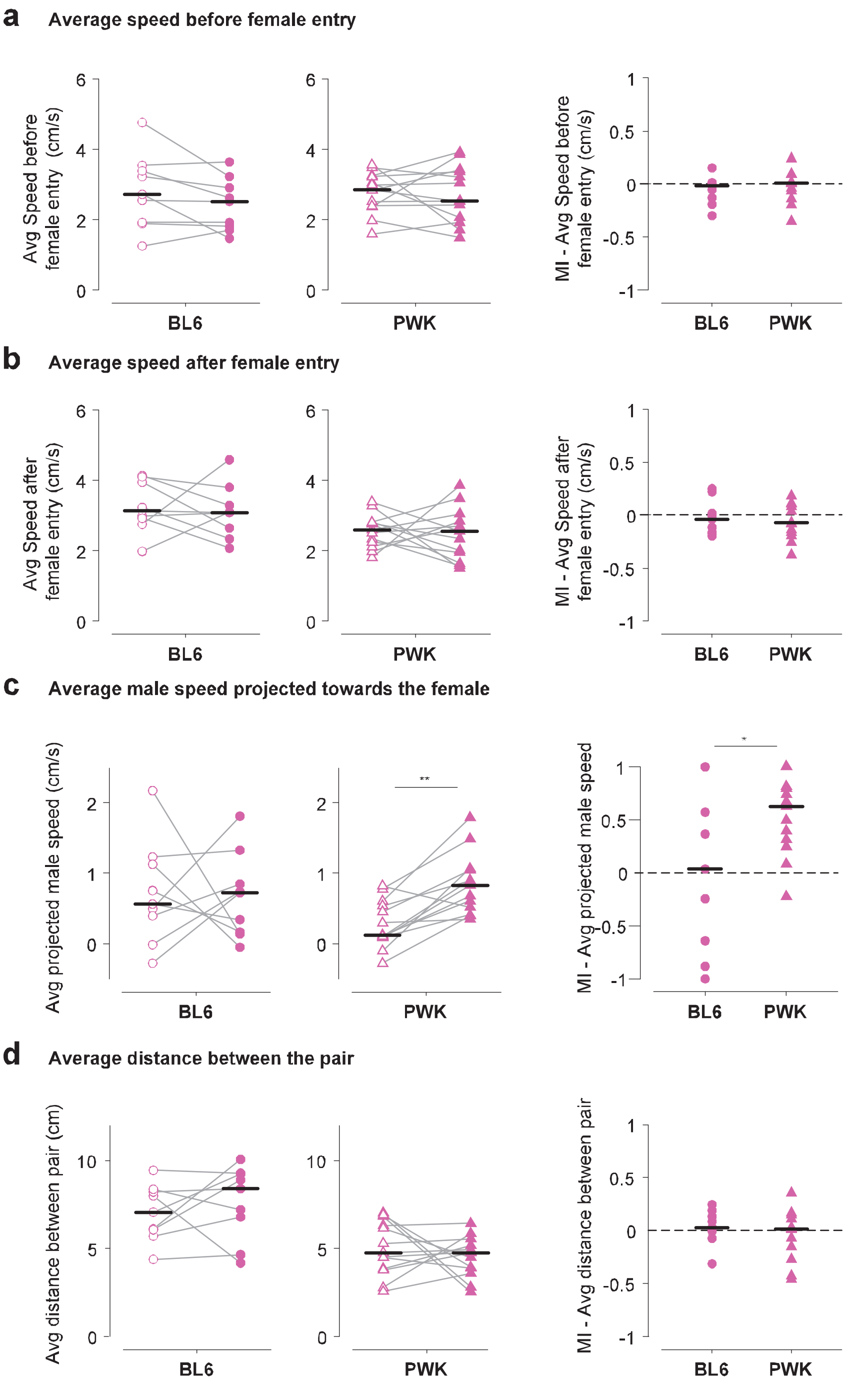

Supplementary Data 3 Bromocriptine treated animals do not show increased locomotion activity. Average speed of the male **a** before and **b** after the female entry until the first consummatory event. **c** Average male speed projected towards the female **d** average distance between the pair. Metrics were taken from couples that reached ejaculation in both sessions from bromocriptine assay BL6 n= 9, PWK n =13 (Fig. 3). Each line represents data of an individual; MI [modulation index (bromo-veh)/(bromo+veh)]. Data presented as median ±

M.A.D. (median absolute deviation with standard scale factor) following Wilcoxon rank sum test. Significance was accepted at $P < 0.05$. * $P < 0.05$, ** $P < 0.01$.

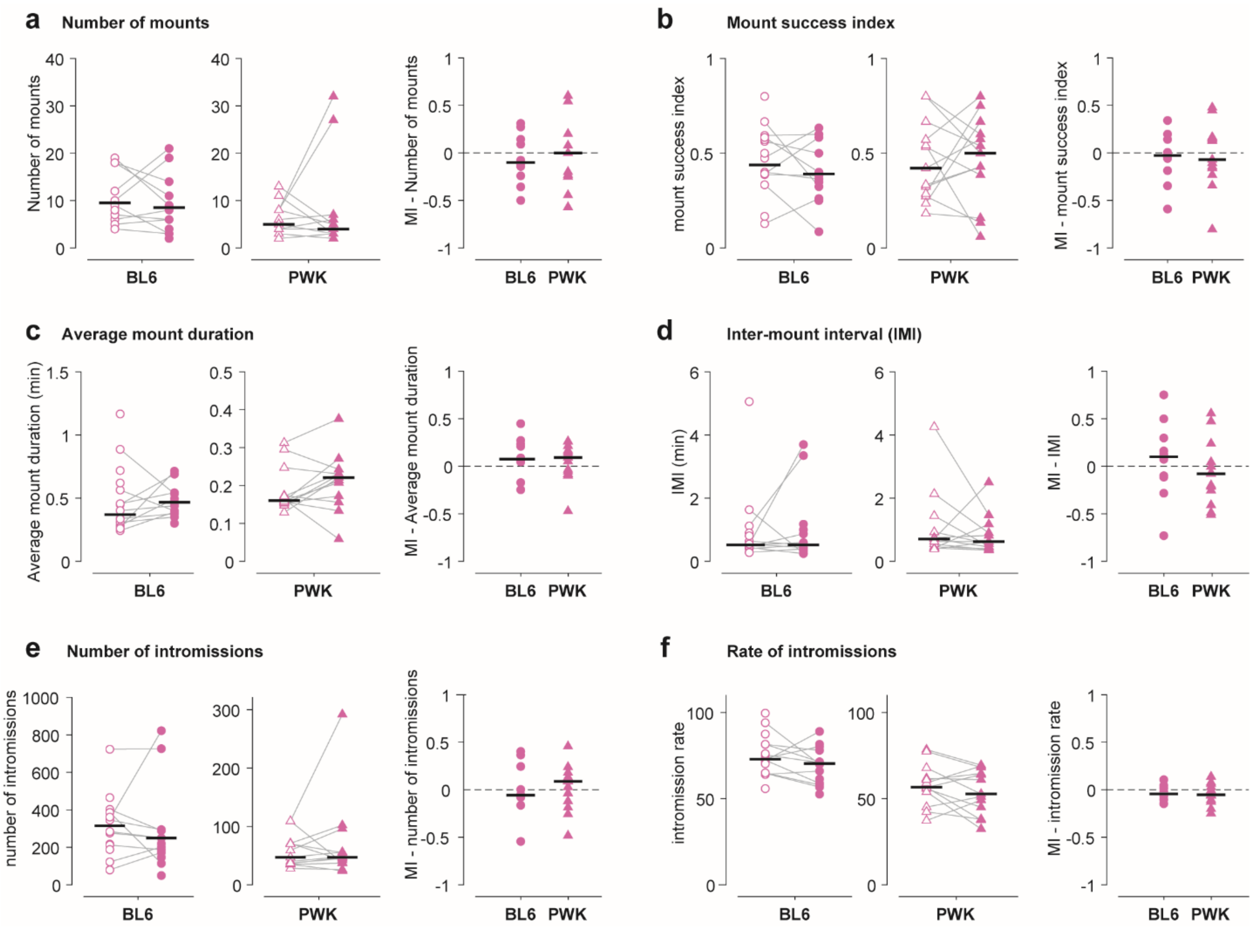

Supplementary Data 4 Additional analysis of the sexual behavior assay using adult sexually trained BL6 and PWK males pre-treated with vehicle or Bromocriptine (total number of subjects used: BL6 n = 18; PWK, n =13; with ejaculation in both sessions BL6 n = 10; PWK, n =13). Quantification of **a** number of mounts, **b** mount success index [mounts/(mount attempt + mounts)], **c** mount mean duration (total mount duration/#mounts), **d** Inter-mount interval (IMI), **e** number of intromissions and **f** rate of intromissions (#intromissions/total mount duration). Each animal underwent two behavioral assays: one with vehicle and one with domperidone. Administrations were contra balanced between animals. Each line represents data of an individual; Unconnected dots represent data of an individual that did not ejaculate in the matching trial (data not used for statistics). MI [modulation index (bromo-veh)/(bromo+veh)]. Data presented as median ± M.A.D. (median absolute deviation with standard scale factor) following Wilcoxon rank sum test. Significance was accepted at $P < 0.05$.
