# Supplementary material for: No evidence for prolactin’s involvement in the post-ejaculatory refractory period
Source: Commun Biol. 2021 Jan 4;4:10. doi: 10.1038/s42003-020-01570-4 (PMC7782750; doi:10.1038/s42003-020-01570-4)
Supplement: Supplementary file 4 — Reporting Summary [file 42003_2020_1570_MOESM4_ESM.pdf]

nature research

Corresponding author(s): Susana Q Lima

Last updated by author(s): 2020/12/04

Reporting Summary

Nature Research wishes to improve the reproducibility of the work that we publish. This form provides structure for consistency and transparency in reporting. For further information on Nature Research policies, see our Editorial Policies and the Editorial Policy Checklist.

Statistics

For all statistical analyses, confirm that the following items are present in the figure legend, table legend, main text, or Methods section.

n/a	Confirmed	
☐	☒	The exact sample size (*n*) for each experimental group/condition, given as a discrete number and unit of measurement
☐	☒	A statement on whether measurements were taken from distinct samples or whether the same sample was measured repeatedly
☐	☒	The statistical test(s) used AND whether they are one- or two-sided<br>*Only common tests should be described solely by name; describe more complex techniques in the Methods section.*
☐	☒	A description of all covariates tested
☐	☒	A description of any assumptions or corrections, such as tests of normality and adjustment for multiple comparisons
☐	☒	A full description of the statistical parameters including central tendency (e.g. means) or other basic estimates (e.g. regression coefficient) AND variation (e.g. standard deviation) or associated estimates of uncertainty (e.g. confidence intervals)
☐	☒	For null hypothesis testing, the test statistic (e.g. *F*, *t*, *r*) with confidence intervals, effect sizes, degrees of freedom and *P* value noted<br>*Give P values as exact values whenever suitable.*
☒	☐	For Bayesian analysis, information on the choice of priors and Markov chain Monte Carlo settings
☒	☐	For hierarchical and complex designs, identification of the appropriate level for tests and full reporting of outcomes
☒	☐	Estimates of effect sizes (e.g. Cohen's *d*, Pearson's *r*), indicating how they were calculated

Our web collection on statistics for biologists contains articles on many of the points above.

Software and code

Policy information about availability of computer code

Data collection	Bonsai (https://bitbucket.org/horizongir/bonsai) was used for data aquisition; idtracker.ai (https://idtracker.ai/en/latest/index.html) was used for determining animal ID and position in the behavioral apparatus.
Data analysis	Behavior was annotated using Python Video Annotator (https://pythonvideoannotator.readthedocs.io). Behavioral data was analyzed using Matlab R2019b and prolactin blood levels were analyzed with GraphPad Prism 7.

For manuscripts utilizing custom algorithms or software that are central to the research but not yet described in published literature, software must be made available to editors and reviewers. We strongly encourage code deposition in a community repository (e.g. GitHub). See the Nature Research guidelines for submitting code & software for further information.

Data

Policy information about availability of data

All manuscripts must include a data availability statement. This statement should provide the following information, where applicable:

- Accession codes, unique identifiers, or web links for publicly available datasets
- A list of figures that have associated raw data
- A description of any restrictions on data availability

All data generated to support the findings of this study are available from the corresponding author upon reasonable request.

Field-specific reporting

Please select the one below that is the best fit for your research. If you are not sure, read the appropriate sections before making your selection.

- [x] Life sciences
- [ ] Behavioural & social sciences
- [ ] Ecological, evolutionary & environmental sciences

For a reference copy of the document with all sections, see nature.com/documents/nr-reporting-summary-flat.pdf

Life sciences study design

All studies must disclose on these points even when the disclosure is negative.

Sample size	Sample size was not predetermined. Sample size was chosen given the design of the study as we used two different mouse strains to validate our results and also because we have within animal sampling, attending to the reduction and refinement policies on animal research.
Data exclusions	Exclusion criteria was pre-established for all experiments. Animals extremely agitated were excluded from the trials.<br>Only males that did 3 ejaculations in 4 mating sessions were selected for the behavioral assays.<br>Data of 1 Bl6 pair was excluded from the idTracker analysis as the tracking system did not perform well due to a video artifact.
Replication	Each experiment was repeated in multiple animals whereby all the results in the paper were obtained from the analysis of multiple animals.<br>For uElisa we had a control sample that was loaded in all plates to guaranty reproducibility between plate and each sample was done in duplicates.<br>Each behavioral assay had its own control as the experiment was within animal control.
Randomization	For prolactin measurements, after training, animals were randomly assigned to each experiment.<br>For the behavioral assays each animal underwent two behavioral sessions: one with vehicle and one with the designated drug. Administrations were counter balanced between animals.<br>All experiments were done in parallel for both strains of mice.
Blinding	The investigator was not blind to group allocation during data collection but was blind during data analysis.

Reporting for specific materials, systems and methods

We require information from authors about some types of materials, experimental systems and methods used in many studies. Here, indicate whether each material, system or method listed is relevant to your study. If you are not sure if a list item applies to your research, read the appropriate section before selecting a response.

Materials & experimental systems

n/a	Involved in the study	
[x]	[ ]	Antibodies
[x]	[ ]	Eukaryotic cell lines
[x]	[ ]	Palaeontology and archaeology
[ ]	[x]	Animals and other organisms
[x]	[ ]	Human research participants
[x]	[ ]	Clinical data
[x]	[ ]	Dual use research of concern

Methods

n/a	Involved in the study	
[x]	[ ]	ChIP-seq
[x]	[ ]	Flow cytometry
[x]	[ ]	MRI-based neuroimaging

Animals and other organisms

Policy information about studies involving animals; ARRIVE guidelines recommended for reporting animal research

Laboratory animals	Mouse: 57BL/6J males and females, 2-6 months of age; PWD/PhJ females, 2-8 months of age; PWK/PhJ males, 2- 6 months of age.
Wild animals	The study did not involve wild animals.
Field-collected samples	The study did not involve samples collected from the field.
Ethics oversight	All experiments were approved by the Animal Care and Users Committee of the Champalimaud Neuroscience Program and the Portuguese National Authority for Animal Health (Direcção Geral de Veterinária).

Note that full information on the approval of the study protocol must also be provided in the manuscript.
